# Efficacy and Safety of Vildagliptin Versus Other Dipeptidyl Peptidase 4 (DPP-4) Inhibitors in the Management of Type 2 Diabetes Mellitus: A Systematic Review of Randomized Controlled Trials

**DOI:** 10.7759/cureus.91432

**Published:** 2025-09-01

**Authors:** Vinodhini Selvaraj

**Affiliations:** 1 General Practice, Sri Lalithambigai Medical College and Hospital, Chennai, IND

**Keywords:** dpp-4 inhibitors, fasting plasma glucose, postprandial glucose, sitagliptin, vildagliptin

## Abstract

Dipeptidyl peptidase 4 inhibitors (DPP-4 inhibitors) are used as second-line drugs in the treatment of type 2 diabetes mellitus (T2DM) patients. They act by preventing the breakdown of incretin hormones, which enhance insulin secretion and reduce glucagon secretion. Vildagliptin and sitagliptin are more commonly used DPP-4 inhibitors. In recent years, the use of DPP-4 inhibitors has been increasing; hence, it is important to evaluate the comparative efficacy and safety of this medication with available evidence. Moreover, this systematic review will evaluate to look for any specific superiority or safety advantage of using Vildagliptin over other DPP-4 inhibitors.

In accordance with Preferred Reporting Items for Systematic Reviews and Meta-Analyses (PRISMA) guidelines, we conducted a systematic review search with Cochrane and PubMed databases. Two independent reviewers examined randomized controlled trial (RCT) studies against the inclusion criteria. Excluded studies involve type 1 diabetes, gestational diabetes, and severe acute diabetic complications. Finally, five RCT studies were chosen, involving 296 participants overall. Baseline and outcome values with p-values and intergroup differences with their p-value from original studies were gathered to evaluate the significance of using vildagliptin over DPP-4 inhibitors, and findings were provided in a narrative way with available evidence.

All five RCT studies have demonstrated a significant reduction in HbA1c from baseline, ranging from -0.3 to -1.34 with p-value <0.05 in the vildagliptin group and -0.1 to -1.07 with p-value >0.05 in other DPP-4 inhibitors, with no significant intergroup differences indicating comparable efficacy between vildagliptin and other DPP-4 inhibitors. Similarly, no significant intergroup differences were observed between vildagliptin and comparator agents in reducing fasting plasma glucose and postprandial glucose. Notably, one study reported a significant reduction favoring vildagliptin, while another showed a greater reduction of FPG with alogliptin; however, intergroup comparisons were not statistically significant. In addition, vildagliptin did not show consistent improvement in lipid profile across the involved studies. Vildagliptin showed a low incidence of hypoglycemic events in comparison with other DPP-4 inhibitors.

Overall, this systematic review found no significant superiority of vildagliptin over other DPP-4 inhibitors such as sitagliptin and alogliptin in the management of type 2 diabetes mellitus, whether used as monotherapy or in combination therapy.

## Introduction and background

Type 2 diabetes mellitus (T2DM) is one of the common types of diabetes noted across the world and accounts for 90% as per the International Diabetes Federation [1,2]. It is characterized by impaired insulin secretion and insulin resistance, where the body will not fully respond to insulin. T2DM is diagnosed by an HbA1c level of ≥6.5% or a fasting plasma glucose (FPG) level of ≥126 mg/dL (≥ 7mmol/L) [3-6]. As per the International Diabetes Federation (2025), 11.1% (589 million adults) or 1 in 9 of the adult population (20-79 years old) are living with diabetes currently, which is expected to reach 1 in 8 by 2050 (853 million adults) [2]. Diabetes contributes to 4.6 million deaths annually. Diabetic patients in the earlier age group had more pronounced beta-cell dysfunction and were at high risk of developing complications earlier [6-9]. Diabetes can lead to multiple micro and macrovascular complications like diabetic neuropathy, nephropathy, retinopathy, cardiovascular disease, foot ulcer, amputation, and depression [10-13], altogether leading to poor quality of life, increased mortality, and morbidity. Therefore, the management of diabetes has significantly progressed beyond strict diabetic control to address the wider complications associated with the disease, including cardiovascular disease and chronic kidney disease.

There are currently multiple conventional drugs available for the management of T2DM, like metformin, sulfonylureas, DPP-4 inhibitors, sodium glucose cotransporter 2 inhibitors, glucagon-like peptide 1 agonists (GLP-1 agonists), thiazolidinediones, and insulin [11-15]. Drugs like DPP-4 inhibitors and GLP-1 agonists are based on the incretin system. DPP-4 is an important enzyme involved in the metabolism of glucose, by degradation of glucagon-like peptide-1 and glucagon-dependent insulinotropic polypeptide (GIP) [14,16]. These gut-derived hormones are secreted after food intake. Hence, these medications work through increasing the availability of endogenous incretin hormones like glucagon-like peptide-1 and GIP, which enhance glucose-dependent insulin secretion and suppress glucagon secretion [14,16-19]. GLP-1 agonists are mostly available in injectable form, so they have poor adherence among patients, whereas DPP-4 inhibitors are in oral form.

Currently, DPP-4 inhibitors are used as a second line of drugs in patients on metformin or sulfonylureas [10,12]. In recent years, the American Diabetes Association and the European Association for the Study of Diabetes have recommended DPP-4 inhibitors to be used as first-line medication in type 2 diabetic patients where metformin is contraindicated or not well tolerated, or in a dual or triple agent regimen [16,19]. There are multiple systematic reviews on sitagliptin, linagliptin, saxagliptin, and alogliptin, whereas a systematic review on vildagliptin is comparatively less. In addition, sitagliptin, linagliptin, saxagliptin, and alogliptin were FDA-approved drugs, whereas vildagliptin was approved by the European Medicine Agency but not by the FDA. Hence, this systematic review aims to analyze and find the overall efficacy of superiority of vildagliptin over other DPP-4 inhibitors in reducing blood glucose level, HbA1c, improving lipid profile, and safety profile of the medication based upon available randomized clinical trials.

## Review

Methods

This systematic review was conducted in accordance with the Preferred Reporting Items for Systematic Reviews and Meta-Analyses (PRISMA) 2020 guidelines [15].

Data search strategy

A comprehensive literature search was conducted on 1st of June 2023, using two electronic databases: PubMed and the Cochrane Library. The search included publications from 1st January 2010 to 30th June 2023. Search terms included a combination of MeSH terms and keywords: “diabetes mellitus,” “type 2 diabetes mellitus,” “DPP-4 inhibitor,” “Dipeptidyl peptidase 4 inhibitors,” “vildagliptin,” “sitagliptin,” “linagliptin,” “alogliptin,” “HbA1c,” “blood glucose levels,” and “adverse events. “PubMed: ((vildagliptin) AND (diabetes mellitus)); Cochrane: diabetes mellitus in All Text AND DPP-4 inhibitor in Title/Abstract/Keyword AND Vildagliptin in Title/Abstract/Keyword (including word variations). Filters applied are Human studies, English language, Clinical trials only, Free full-text.

Cochrane and PubMed are internationally recognized and provide a wide range of randomized controlled trials (RCTs), while other databases like Scopus and Embase may provide duplicate studies and low-quality studies. Hence, in order to avoid duplication, non-peer-reviewed and poor-quality articles, we have included studies from these two databases.

Inclusion criteria

Participants

Participants were adults diagnosed with T2DM, receiving lifestyle modifications or metformin or other antidiabetic medications.

Intervention

Intervention used was DPP-4 inhibitor therapy - vildagliptin.

Comparator

Comparison with other DPP-4 inhibitors like sitagliptin and alogliptin was done.

Outcomes

Changes in HbA1c, FPG, postprandial blood glucose (PPBG), LDL, HDL, and triglyceride levels were assessed. In addition, the safety profile of DPP-4 inhibitors was assessed.

Study Design

The study design was primary research, including randomized controlled trials or clinical trials.

Duration of the Study

The duration of the study was ≥ 8 weeks. Clinical trials have consistently shown that significant improvements in glycemic markers were observed as early as eight weeks after starting therapy. Therefore, an eight-week duration is sufficient to evaluate the initial efficacy and tolerability of vildagliptin compared to other agents [4-6].

Other Criteria

Other criteria include only studies involving human subjects and published in English.

Exclusion criteria

Non-primary research such as systematic reviews, narrative reviews, case reports, or case series was excluded. Studies involving participants with type 1 diabetes mellitus, gestational diabetes mellitus, severe acute diabetic complications like diabetic ketoacidosis or hyperosmolar state were also excluded [14].

Outcomes evaluated

This systematic review focuses on assessing glycemic control, metabolic outcomes and adverse events associated with DPP-4 inhibitors, with the aim on whether vildagliptin demonstrates comparable efficacy with other medications in the same group. Primary outcomes were HbA1C as per Zhou et al. (2019) [20]. Secondary outcomes were FPG, PPBG, LDL, HDL, triglyceride level, and incidence of adverse events.

Study selection and reviewing process

**Figure 1:**
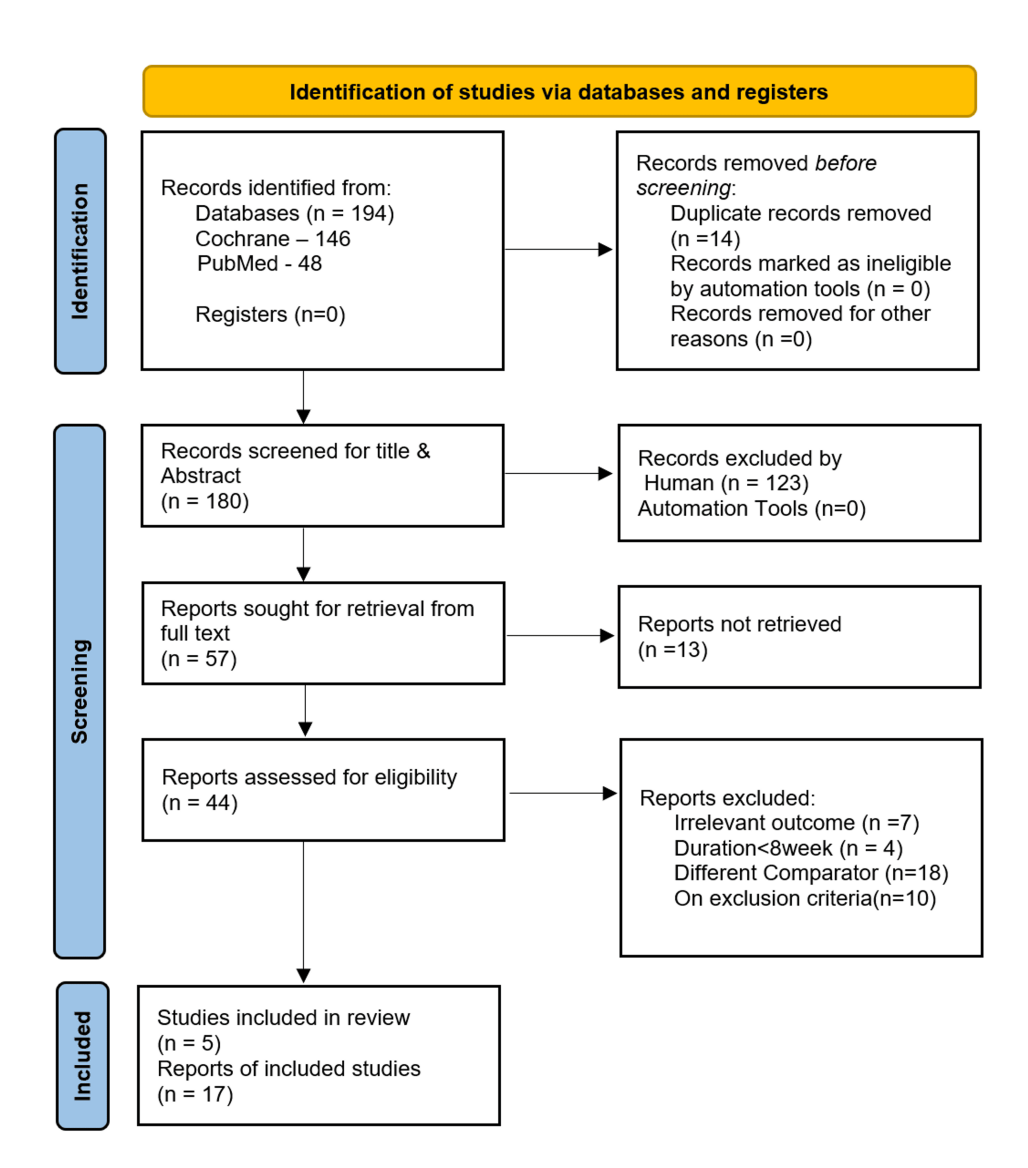
PRISMA flow chart PRISMA: Preferred Reporting Items for Systematic Reviews and Meta-Analyses

Two reviewers scrutinized the articles separately as per pre-defined inclusion and exclusion criteria. The screening process was conducted independently by two reviewers. Initially, titles and abstracts were evaluated to eliminate irrelevant studies, followed by full-text evaluation to confirm eligibility. Only studies with open access to full-text articles were considered for full-text review. Any disagreements were resolved through consensus. The study selection process is illustrated in the PRISMA 2020 flow diagram (Figure 1).

Data extraction

Two reviewers took part independently in data extraction. Initially, one reviewer examined all the citations for potential eligibility, and the other reviewer looked into the excluded articles to ensure accuracy. Thereafter, full-text articles were reviewed independently by two reviewers. For eligible studies, following information was extracted by one reviewer: First author name, year, study design, blinding and bias control, patient information (mean age, duration of T2DM, baseline HbA1c, eGFR), medication name, dose, duration of study, comparator medication information, primary and secondary outcomes like HbA1c, FBG, PPBG, LDL, HDL, triglycerides, and adverse events. The other reviewer verified the data gathered. Any discrepancies are sought by mutual agreement.

Since the included studies show heterogeneity in study duration, population characteristics, outcome, and reported formats, pooled meta-analysis can lead to misleading conclusions. Therefore, we planned to extract baseline and outcome values with p-values as well as intergroup difference and its p-value from original studies to evaluate the significance of using vildagliptin over DPP-4 inhibitors, and to provide a narrative summary of findings in a clear and transparent way with available evidence. p-value <0.05 was considered to be significant.

Risk of bias assessment

Using the JADAD scoring system [11], a validated tool for evaluating methodological quality in RCTs, two reviewers individually looked at all the included RCTs for risk of bias. They focused on three core domains: (i) Randomization process, (ii) Blinding of participants and personnel, and (iii) Reporting of withdrawals and dropouts of all patients. The JADAD tool has been chosen for its simplicity and effectiveness in detecting major sources of bias in clinical trials [11]. Each study received a JADAD score ranging from 0 to 5, with higher scores of ≥ 3 indicating greater methodological quality. Disagreements in scoring were discussed and resolved through consensus.

Results of study selection

A comprehensive literature search was conducted following the PRISMA 2020 guidelines [15]. An initial search across two major databases yielded 194 articles. After removing 14 duplicates using EndNote, 180 records remained for title and abstract screening. Of these, 123 articles were excluded due to irrelevance to the research question. Thereafter, eliminated articles for following reasons: 13 articles did not provide open access to full-text, seven studies reported outcomes that were not relevant to the review, four studies had a treatment duration of less than eight weeks, 18 studies used comparators outside the scope of inclusion criteria (e.g., placebo, SGLT2 inhibitors, GLP-1 agonists, thiazolidinediones, insulin), and 10 studies involved patients with severe diabetic complications, thus meeting exclusion criteria. After applying all inclusion and exclusion criteria, five RCTs were deemed eligible and included in the final synthesis. All selected studies were RCTs and met the methodological and clinical criteria for inclusion. In total, 17 reports were extracted from the five selected RCTs. The detailed characteristics of studies are illustrated in Table 1.

**Table 1:** Characteristics of included studies with Jadad scoring RCT: randomized control trial; T2DM: type 2 diabetes mellitus; OHAs: oral hypoglycemic drugs; DPP4 inhibitor: dipeptidyl peptidase 4 inhibitor

Author & Date	Study Design	Country	Number of Subjects in Treatment/Control Group	Patient Characteristics	Mean Age in Treatment/Control Group	Treatment Dose	Control or Comparator Dose	Follow-Up	Jadad Score
Elhini et al., 2021 [7]	RCT	Egypt	20/20	Newly diagnosed type 2DM not on OHA	48.3/49.1	Vildagliptin 50mg twice daily	Sitagliptin 100mg once daily	12 weeks	2
Guerci et al., 2012 [8]	RCT	France	19/19	T2DM on metformin, willing to undergo continuous glucose monitoring for 3 days	59.1/53.5	Vildagliptin 50mg twice daily	sitagliptin 100mg once daily	8 week	3
Koyanagawa et al., 2016 [12]	RCT	Japan	17/16	T2DM on sitagliptin 50mg/day and insulin	65.9/64.5	Vildagliptin 50mg twice daily	sitagliptin 100mg once daily	8 weeks	3
Li et al., 2014 [14]	RCT	China	69/68	T2DM with dual combination of metformin and other traditional oral hypoglycemic agents (glimepiride, acarbose and pioglitazone).	44.8/48.6	Vildagliptin 50mg twice daily	Sitagliptin 100mg once daily	24 weeks	3
Tanaka et al., 2017 [19]	RCT	Japan	23/25	T2DM on OHA but not on DPP-4 inhibitor	65.8/63.6	Vildagliptin 50mg twice daily	Alogliptin 25mg /day	24 week	3

Patient characteristics

The baseline characteristics of the five RCTs included in this systematic review are illustrated in Table 1. All the selected studies had evaluated vildagliptin in comparison with other DPP-4 inhibitors, with most trials being open-label and randomized in design. One of the included studies (Elhini et al., 2021) was a pilot trial [7].

The duration of interventions ranged from 8 to 24 weeks. All five studies directly compared vildagliptin with other DPP-4 inhibitors [7,8,12,14,19]. Sitagliptin served as a comparator in four studies: Elhini et al. (2021) [7]; Guerci et al. (2012) [8]; Koyanagawa et al. (2016) [12]; and Li et al. (2014) [14], collectively involving 123 participants in the comparator groups. Alogliptin was used as the comparator in one study by Tanaka et al. (2017) [19], involving 25 participants. In total, 296 participants with T2DM were enrolled across all five studies, with 148 patients allocated to vildagliptin.

The mean age group of participants across studies ranged from 44 to 65 years. All studies included participants with HbA1c levels between 7% and 11% as part of their inclusion criteria. Elhini et al. (2021) [7] and Tanaka et al. (2017) [19] included patients with HbA1c levels of more than 6.5%, while Li et al. (2014) [14] restricted enrollment to those with HbA1c values between 7.5% and 10%. Koyanagawa et al. (2016) [12] included participants with HbA1c levels between 6.2% and 8.4%, and Guerci et al. (2012) [8] selected patients with values between 6.5% and 8%. The primary outcome in three of the studies was reduction in HbA1c (Guerci et al., 2012; Li et al., 2014; Tanaka et al., 2017) [8,14,19], whereas in two studies (Elhini et al., 2021; Koyanagawa et al., 2016), HbA1c was reported as a secondary outcome [7,12].

Due to heterogeneity of included studies, findings were narratively summarized rather than choosing meta-analysis in order to avoid a misleading conclusion.

HbA1c reduction

All five studies have shown HbA1C as the primary outcome. Two studies with a short duration of eight weeks reported significant reductions of HbA1C from baseline ranging from -0.3% (p = 0.006) to -0.5% (p < 0.001) in the vildagliptin group, while the reduction in the sitagliptin group was not statistically significant 0.1% -0.3% p >0.05 [8,12]. A 12-week trial by Elhini et al. (2021) [7] revealed substantial HbA1c reductions in both groups; vildagliptin showed the highest mean reduction of HbA1C -1.02% with p = 0.0001 from baseline and -0.96% (p = 0.0001) for sitagliptin. Furthermore, two 24-week studies comparing vildagliptin with either sitagliptin or alogliptin showed statistically significant reductions in both treatment groups, highest ranging from -0.7% to -1.34% (p = 0.001) in the vildagliptin group compared to -0.5% to -1.07% (p = 0.002) in comparator groups [14,19]. All five RCT studies have demonstrated a significant reduction in HbA1c from baseline with vildagliptin treatment. Across all included studies, no significant intergroup differences were observed, indicating comparable efficacy between vildagliptin and other DPP-4 inhibitors in HbA1c reduction (as shown in Table 2).

**Table 2:** Results of each study selected for this systematic review T: treatment drug; C: comparator drug; P: p-value; FPG: fasting plasma glucose; PPBG: postprandial blood glucose; Tg: triglyceride, LDL: low-density lipoprotein; HDL: high-density lipoprotein.

Author & Date	Follow-up	Mean Reduction in HbA1c From Baseline % Outcome 1			Mean Reduction in FPG (mg/dl) Outcome 2			Mean Reduction in PPBG (mg/dl) Outcome 3			Mean reduction in LDL (mg/dl) Outcome 4			Mean reduction in TG (mg/dl) Outcome 5			Mean increase in HDL (mg/dl) Outcome 6		
		T	C	P	T	C	P	T	C	P	T	C	P	T	C	P	T	C	P
Guerci et al., 2012 [8]	8 weeks	-0.5	-0.3	0.42	-15	-15	0.73	-	-	-	-	-	-	-	-	-	-	-	-
Koyanagawa et al., 2016 [12]	8 weeks	-0.3	-0.1	0.15	-	-	-	-	-	-	-0.1	-1.3	0.88	-20	-12	0.26	0.9	0.2	0.92
Elhini et al., 2021 [7]	12 weeks	-1.02	-0.96	0.41	-89.6	-69.9	0.011	-140.1	-121.8	0.13	-34.3	-31.9	0.451	-36.4	-22.8	0.0001	2.85	5.25	0.000
Li et al., 2014 [14]	24 weeks	-1.34	-1.07	0.26	-43.92	-26.8	1.26	-66.78	-56.88	1.17	-	-	-	-	-	-	-	-	-
Tanaka et al., 2017 [19]	24 week	-0.7	-0.5	0.219	-6.1	-16.6	0.513	-	-	-	-5.9	-3.5	0.144	-0.4	-1.2	0.279	17.7	12.8	0.209

Fasting plasma glucose 

FPG reduction was assessed in four out of five included studies. The study by Li et al. (2014) [14] reported FPG values in mmol/L, which were converted to mg/dL for consistency. Tanaka et al. (2017) [19] observed a greater reduction in the alogliptin group (-16.6 mg/dL, p = 0.016) compared to vildagliptin (-6.1 mg/dL, p = 0.344) with insignificant intergroup difference (p-value -0.513). To the contrary, only the study by Elhini et al. (2021) [7] found a significant intergroup difference, with a superiority of vildagliptin over sitagliptin (-21.7 mg/dL, p = 0.011).

Overall, three studies observed statistically insignificant intergroup comparison between vildagliptin and comparator group (sitagliptin or alogliptin) in reducing the fasting glucose (see Table 2) with only one study [7] observed a significant intergroup difference and other study [19] observed a greater reduction in the alogliptin group (-16.6 mg/dL, p = 0.016) compared to vildagliptin (-6.1 mg/dL, p = 0.344), with insignificant intergroup comparison.

Postprandial blood glucose 

Only two studies assessed PPBG reduction. Elhini et al. (2021) [7] demonstrated a statistically significant reduction in PPBG in both the vildagliptin group (-140.1, p-value = 0.0001) and sitagliptin group (-121.8, p-value = 0.0001), with an insignificant intergroup difference (p-value= 0.13). Conversely, the study by Li et al. (2014) [19] reported non-significant reductions in both treatment arms, with no meaningful intergroup difference (0.55). Altogether, both the studies showed no significant superiority of vildagliptin to sitagliptin.

LDL, triglyceride, and HDL level changes

Three studies [7,12,19] evaluated the effects of vildagliptin on lipid parameters, LDL, TG, and HDL levels. Two studies [12,19] reported no significant improvement in lipid levels across all groups (vildagliptin, sitagliptin, and alogliptin), with both intragroup and intergroup comparisons showing no statistically significant differences in lipid parameters. Conversely, Elhini et al. (2021) [7] reported significant reductions in LDL and TG levels and an increase in HDL for both the vildagliptin and sitagliptin groups. Furthermore, this study found a significant intergroup difference, with a superiority of vildagliptin over sitagliptin in improving HDL (p-value <0.001) and reducing TG (p-value <0.0001). These results suggest that while vildagliptin may have a modest beneficial effect on lipid profiles, this is not consistently observed across all studies.

Safety outcome

Adverse events were reported in three of the included studies (summarized in Table 3). The overall incidence of adverse events was comparable between the vildagliptin and comparator groups. Specifically, the incidence of hypoglycemia was reported as:7.1% in the vildagliptin group vs 15.1% in the sitagliptin group (Guerci et al., 2012) [8], 2% in the vildagliptin group vs 3% in the sitagliptin group (Li et al., 2014) [14].

**Table 3:** Safety outcome of each selected study This table represents key characteristics of the five studies included in this systematic review, focusing on follow-up duration and reported number of adverse events in both treatment and comparator groups.

Author & Date	Follow-up	Total Number of Adverse Events	
		Treatment n, %	Comparator n,%
Guerci et al., 2012 [7]	8 weeks	11(57.9%)	11(57.9%)
Li et al., 2014 [14]	24 weeks	13(21%)	20 (32%)
Tanaka et al., 2017 [19]	24 weeks	0 (0%)	1(4%)
Koyanagawa et al., 2016 [12]	8 weeks	N/A	N/A
Elhini et al., 2021 [7]	12 weeks	N/A	N/A

No severe adverse events were reported, and all treatments were generally well tolerated. These findings suggest that vildagliptin has a favorable safety profile, with low risk of hypoglycemia and similar overall tolerability to other DPP-4 inhibitors.

Jadad scoring

Due to the limited number of RCTs directly comparing vildagliptin with other DPP-4 inhibitors, all five included studies were open-label trials, and one was classified as a pilot study [9]. The Jadad score [11], used to assess methodological quality, shows 3/5 in four studies, indicating high-quality trials, and 2/5 in one study, suggesting lower quality and potential risk of bias. Despite their open-label nature, the majority of the studies demonstrated sufficient methodological rigor, lending moderate to high confidence to the outcomes assessed. The final scores are presented in Table 4.

**Table 4:** Jadad scoring This table summarizes the methodological quality of the included randomized controlled trials based on the Jadad scoring system, which evaluates the risk of bias related to randomization, blinding, and accountability of all patients.

Author & Date	Randomization	Blinding	An Account of All The Patients	JADAD Score	Quality
Guerci et al.., 2012 [8]	2	0	1	3	High
Koyanagawa et al., 2016 [12]	2	0	1	3	High
Elhini et al., 2021 [7]	1	0	1	2	Moderate
Li et al., 2014 [14]	2	0	1	3	high
Tanaka et al., 2017 [19]	2	0	1	3	High

Discussion

DPP-4 inhibitors are widely used second-line antidiabetic medication. Although sitagliptin and vildagliptin are the same class of drugs, they have different mechanisms of action. Sitagliptin acts as a competitive inhibitor of DPP-4 enzyme, whereas vildagliptin functions as a substrate that forms a covalent complex with the catalytic site of DPP-4, leading to slow dissociation and sustained enzyme inhibition [3,5]. Even though vildagliptin has a short half-life (2-3hours), its mechanism of action will exert prolonged DPP-4 inhibition, resulting in elevated levels of intact glucagon-like peptide-1 (GLP-1), even during fasting states [1]. Consequently, it helps to suppress glucagon secretion and reduces overnight production of hepatic glucose [1-5].

This systematic review aimed to assess the comparative efficacy and safety of vildagliptin versus other DPP-4 inhibitors (sitagliptin, alogliptin), either as monotherapy or in combination therapy, in patients with T2DM with inadequate glycemic control. Key efficacy parameters assessed included changes from baseline in HbA1c, FBG, PPBG, and lipid profile (LDL, HDL, triglycerides), along with adverse event profiles.

Across the five studies included in this review, vildagliptin demonstrated comparable efficacy to other DPP-4 inhibitors in reducing HbA1c. Notably, one study found alogliptin to be superior to vildagliptin in reducing FPG levels [19]. These findings align with previous systematic reviews comparing vildagliptin to other antidiabetic agents [4]. The study by Li et al. [14], which assessed vildagliptin, sitagliptin, and saxagliptin as add-on therapy to dual oral antidiabetic treatment, reported similar proportions of patients achieving HbA1c <7% (vildagliptin: 65%, sitagliptin: 59%, saxagliptin: 59%). A similar trend was noted in the study by Zhou et al. (2019) [20], which has also reported equivalent efficacy across this medication.

In terms of lipid metabolism, vildagliptin has been shown to reduce fasting lipolysis and triglyceride accumulation in non-adipose tissues [1]. Given that LDL cholesterol is a significant contributor to cardiovascular risk in T2DM [17], improvements in lipid parameters could theoretically enhance long-term outcomes. Through these combined mechanisms, like improving glycemic parameters, lipid profile, and blood pressure, vildagliptin exerts a positive effect on metabolic syndrome and can also decrease cardiovascular risk in T2DM patients. However, in this review, only one study demonstrated a statistically significant improvement in lipid profile parameters with vildagliptin [7], while other studies did not show significant changes. Therefore, while vildagliptin can offer certain metabolic benefits, evidence remains limited regarding its effect on changes in lipid metabolism.

Regarding safety, the incidence of adverse events with vildagliptin was mild and comparable to that of other DPP-4 inhibitors such as sitagliptin and alogliptin. Importantly, vildagliptin was associated with a lower risk of hypoglycemia [8,14,19]. This observation aligns with earlier studies that reported a favorable safety profile for vildagliptin, including lower incidence of gastrointestinal side effects and minimal risk of hypoglycemia [20,21]. On the other hand, sitagliptin has been associated with moderate rates of adverse effects such as abdominal discomfort, leukocytosis, and elevated liver enzymes [18].

Collectively, the data suggest that vildagliptin is an effective and well-tolerated DPP-4 inhibitor with glycemic efficacy comparable to other agents in its class. While its impact on lipid profiles remains inconsistent, its unique mechanism of action and favorable safety profile, particularly regarding hypoglycemia, make it a viable option for T2DM management.

Limitations

Like many systematic reviews, this study has several limitations. Firstly, the inclusion of open-label trials introduces potential for performance and detection bias. Secondly, there is a possible risk of selection bias due to variability in participant characteristics, settings, and study designs across included trials, which limits the ability to statistically pool results and estimate the overall effect size. There was a limited number of RCTs available that directly compared vildagliptin with other DPP-4 inhibitors. One among the included studies was a pilot trial, which may limit the generalizability of the findings, and the overall sample size was relatively small, reducing the statistical power and robustness of the conclusions. In addition, concomitant use of other antidiabetic medications such as metformin and sulfonylureas might have confounded the observed effects of the DPP-4 inhibitors.

Our literature search was conducted up to 30th June 2023 in accordance with the predefined protocol. Studies published after this cut-off were not included to maintain consistency with our search strategy and systematic analysis. Moreover, we have spent considerable time screening, critical appraisal and statistical analysis of this systematic review, in order to ensure methodological rigor. The preparation of a high-quality manuscript, including writing, formatting, internal review, and revisions, naturally extends over several months. We acknowledge that additional studies have been published after our search cut-off period and they were not included in the present review but will be considered in future updates of this work.

## Conclusions

In summary, this systematic review highlights the efficacy and safety of DPP-4 inhibitors, particularly vildagliptin, in improving glycemic and metabolic outcomes in patients with T2DM. This review found no significant differences in efficacy between vildagliptin over other DPP-4 inhibitors, such as sitagliptin and alogliptin, in the management of T2DM, whether used as monotherapy or in combination therapy. Vildagliptin demonstrated comparable effectiveness with other DPP-4 inhibitors in reducing HbA1c, FPG, and PPBG and in improving lipid profiles, with a low incidence of hypoglycemic events. However, further high-quality, long-term studies are needed to fully evaluate the sustained efficacy and safety of vildagliptin in diverse patient populations.
